# Income inequality is associated with adolescent fertility in Brazil: a longitudinal multilevel analysis of 5,565 municipalities

**DOI:** 10.1186/s12889-015-1369-2

**Published:** 2015-02-07

**Authors:** Alexandre DP Chiavegatto Filho, Ichiro Kawachi

**Affiliations:** University of São Paulo, Av Dr. Arnaldo, 715 01255-000 São Paulo, Brazil; Harvard University, Boston, MA, USA

## Abstract

**Background:**

Brazil has one of the highest adolescent fertility rates in the world. Income inequality has been frequently linked to overall adolescent health, but studies that analyzed its association with adolescent fertility have been performed only in developed countries. Brazil, in the past decade, has presented a rare combination of increasing per capita income and decreasing income inequality, which could influence future desirable pathways for other countries.

**Methods:**

We analyzed every live birth from 2000 and from 2010 in each of the 5,565 municipalities of Brazil, a total of 6,049,864 births, which included 1,247,145 (20.6%) births from women aged 15 to 19. Income inequality was assessed by the Gini Coefficient and adolescent fertility by the ratio between the number of live births from women aged 15 to 19 and the number of women aged 15 to 19, calculated for each municipality. We first applied multilevel models separately for 2000 and 2010 to test the cross-sectional association between income inequality and adolescent fertility. We then fitted longitudinal first-differences multilevel models to control for time-invariant effects. We also performed a sensitivity analysis to include only municipality with satisfactory birth record coverage.

**Results:**

Our results indicate a consistent and positive association between income inequality and adolescent fertility. After controlling for per capita income, college access, youth homicide rate and adult fertility, higher income inequality was significantly associated with higher adolescent fertility for both 2000 and 2010. The longitudinal multilevel models found similar results. The sensitivity analysis indicated that the results for the association between income inequality and adolescent fertility were robust. Adult fertility was also significantly associated with adolescent fertility in the cross-sectional and longitudinal models.

**Conclusion:**

Income inequality is expected to be a leading concern for most countries in the near future. Our results suggest that changes in income inequality are positively and consistently associated with changes in adolescent fertility.

## Background

Brazil has one of the highest adolescent fertility rates in the world. According to a report by the World Health Organization (WHO) [1], it is one of seven countries that account for half of all adolescent fertility (along with Bangladesh, the Democratic Republic of Congo, Ethiopia, Nigeria, India and the United States). From 2000 to 2010, there were 6,829,940 live births among women aged 15 to 19 in Brazil, corresponding to 20.7% of total births in the country.

Various theories have been put forward to account for adolescent pregnancies. Most focus on individual factors such as the absence of sex education and the availability of contraceptive measures [2,3]. More recent theories have focused on contextual factors, such as the local perception of (lack of) opportunities for economic advancement, frequently included within the framework of a “culture of despair” [4]. According to this theory, adolescents faced with an unequal and segregated society are de-motivated from investing in their own human capital (i.e. staying in school), leading to the anticipation of motherhood choices [5].

Income inequality is a growing source of concern for most developed countries, as the disparity between rich and poor individuals has been consistently growing since the last decades of the 20^th^ century [6]. It is frequently measured by the Gini Coefficient, a measure of income dispersion that varies from zero, expressing perfect equality, up to one, meaning that just one individual receives all the income. In 2010, the Gini Coefficient for developed countries, according to the The Organisation for Economic Co-operation and Development (OECD), was 0.32, while for Brazil it was 0.54 (i.e. more unequal) [7,8].

A recent review of the determinants of adolescent health, part of a special series on adolescents by The Lancet [9], included income inequality as one of the three most important structural factors influencing overall adolescent health (along with educational access and national wealth). According to the authors, “there is substantial evidence that income inequality within countries affects various aspects of adolescent health”.

Despite growing evidence that income inequality affects overall adolescent health, there is a limited number of studies that have analyzed the specific relationship between income inequality and adolescent fertility, all of which used data from developed countries. An ecological study based in 39 US states found an association between income inequality and adolescent fertility, but the association was substantially explained by the correlation between income inequality and poverty [10]. Another study, also using data from the US, found poverty and income inequality to be independently associated with adolescent fertility [11]. A third study analyzed a sample of developed countries and found a cross-sectional association between income inequality and adolescent births [12].

Brazil has historically presented one of the highest levels of income inequality in the world [13]. During the last two decades, however, the country has experienced substantial income growth accompanied by a decrease in income inequality, a rare combination among current economies. From 2000 to 2010, per capita income grew 34% (from R$592.87 in 2000 to R$793.87 in 2010), and income inequality (as measured by the Gini Coefficient) decreased 10%, from 0.60 to 0.54 [8]. Analyzing the health effects of a consistent decrease in income inequality may suggest a future pathway for other countries as they seek to mitigate the adverse consequences of inequality. Accordingly, our study sought to test the association between changes in income inequality and adolescent fertility, by analyzing all live births from the 5,565 municipalities of Brazil for 2000 and 2010.

## Methods

We analyzed every live birth that occurred in the year 2000 and in 2010 (the two years of the last national censuses) in each of the 5,565 municipalities of Brazil, a total of 6,049,864 births-which included 1,247,145 (20.6%) births from women aged 15 to 19 [14]. Our dependent variable of interest was adolescent fertility, calculated by dividing the number of live births among women aged 15 to 19 by the number of female residents aged 15 to 19. Fertility values were presented in terms of live births per 100 adolescents. We used registry data provided by the Ministry of Health (Sistema de Informações sobre Nascidos Vivos, SINASC) [14].

All variables were calculated separately for each municipality. Municipalities in Brazil are the smallest regions with administrative power, encompassing both urban and rural areas, and dividing the entirety of the country - i.e. every area of Brazil is part of a municipality [15]. In general, Brazilian municipalities are quite heterogeneous and right skewed in terms of population, with a median of 10,934 and an average of 34,278 individuals in 2010 [8].

Income inequality was assessed by the Gini Coefficient with data from the United Nations Development Program [16]. The Gini Coefficient uses the Lorenz curve to calculate a value of statistical dispersion that ranges from 0.0 (perfect equality, with every household earning exactly the same) to 1.0 (absolute inequality, with a single household earning the locality’s entire income). It is equivalent to half the average absolute difference between the incomes of any two households randomly sampled for a population, and then normalized to the mean. Gini values ranged from 0.30 (*Alto Feliz*) to 0.87 (*Campos de Júlio*) in the year 2000, and from 0.28 (*São José do Hortêncio*) to 0.80 (*São Gabriel da Cachoeira*) in 2010.

We also included variables that could confound the relationship between income inequality and adolescent health. First, we controlled for the per capita income of the municipality, to take account of the correlation between absolute income and income inequality [17]. Second, we tested if attending college (as measured by percentage of mother with 12 years or more of formal education, or one year more than basic education) could have an effect on delaying childbirth [5]. Third, we included adult female fertility (20 to 49 years old) to test for the presence of an overall change in attitudes toward motherhood [18]. Fourth, we tested if the youth homicide rate (10 to 19 years old) could have an effect in anticipating reproductive decisions [12]. Fifth, we tested for an additional state-level effect of income inequality on adolescent fertility. For the youth homicide rate, we included the results for the three years around the year of reference in order to decrease the influence of random annual variability, an important issue for small municipalities. Therefore, for the homicide rate, the results for the year 2000 referred to the data for 1999, 2000 and 2001, and for 2010 to the data from 2009, 2010 and 2011. Population and birth data were provided by the Ministry of Health, and income and education data from the 2000 and 2010 Brazilian censuses [8,14].

We first tested the association between adolescent fertility and income inequality separately by year (2000 and 2010) by fitting a multilevel regression model. The first level of the model referred to the aggregated results for each municipality. As adolescent pregnancies could be affected by regional factors, we included each of the 27 states of Brazil as the second level of the model to test for a broader cultural influence on adolescent fertility. The first model included only the municipality-level income inequality as the independent variable. The second model added the control variables, i.e. per capita income, college access, adult fertility and youth homicide rate. The third model included state-level income inequality. The same sequence was repeated for 2010. We added a seventh model to test if the association between income inequality and adolescent fertility significantly changed from 2000 to 2010, by including the results from both years (N = 11,130) and adding an interaction term for the year 2010 for all the covariates. For each model, we calculated the deviance of the multilevel model (-2*log-likelihood), a badness-of-fit statistics, where lower values indicate that the model has a better fit to the data.

As adolescent fertility may be affect by other cultural and geographic factors not included in the models, we fitted longitudinal first-difference multilevel models to control for time-invariant effects [19]. We first included all of the 5,565 municipalities, by following the same sequence of inclusion as the previous models. We then performed a sensitivity analysis to test the robustness of the results by including only the municipalities with birth data coverage considered to be satisfactory, assessed by calculating the three-year relative mean deviation of the birth rate of each municipality, with a value over 90% considered as satisfactory [20]. This excluded 3,129 municipalities (56.23% of the total), but only 22.33% of the population. As expected, simple logistic regression indicated that the excluded municipalities were significantly poorer and less educated than the municipalities that had satisfactory birth coverage (data not shown).

For every model, each municipality was weighted by its total population. For models that included 2000 and 2010, municipalities were weighted by the average population for the two years. Descriptive statistics were assessed with Stata 12. Multilevel models were estimated by using MLwiN V.2.29 software.

## Results

Table 1 presents the averages of each of the variables for the years 2000 and 2010. From 2000 to 2010, there was a decrease in average adolescent fertility (from 8.01 to 6.09 per 100 female adolescents) and municipality-level income inequality (from 0.57 to 0.53, as measured by the Gini Coefficient), and an increase in per capita income (from R$586.50 to R$767.36).

**Table 1:** **Descriptive results for the variables included in the analysis**

	**2000**		**2010**	
	**Mean**	**95% CI**	**Mean**	**95% CI**
Adolescent fertility^a^	8.01	7.95; 8.07	6.09	6.04; 6.14
Income inequality^b^	0.57	0.56; 0.57	0.53	0.53; 0.53
Per capita income (R$)	586.50	577.27; 595.74	767.36	756.69; 778.03
Adult fertility^c^	6.46	6.42; 6.51	5.14	5.11; 5.16
Mothers with 12 years or more of formal education (%)	11.68	11.51; 11.86	18.44	18.22; 18.65
Youth homicide rate^d^	3.86	3.76; 3.96	4.05	3.96; 4.14
State-level inequality^b^	0.61	0.61; 0.62	0.58	0.58; 0.58

^a^Ratio between the number of live births from women aged 15 to 19 and the number of women aged 15 to 19.

^b^Calculated by the Gini Coefficient.

^c^Ratio between the number of live births from women aged 20 to 49 and the number of women aged 20 to 49.

^d^Homicide rate (per 10,000) of 10 to 19 years old residents.

Brazilian municipalities, 2000 and 2010.

Table 2 and Table 3 show the results for the cross-sectional multilevel analyses. We first analyzed the results for the year 2000 (Table 2). The first model indicates a positive association between income inequality and adolescent fertility - i.e. higher income inequality was associated with higher adolescent fertility. After including the other municipal-level variables (Model 2), the association between income inequality and adolescent fertility remained statistically significant (p < 0.05). The beta coefficient of 3.23 (95% CI: 1.76; 4.70) for Model 2 indicates that an increase of 0.1 in the Gini coefficient is associated with 0.32 more live births for every 100 adolescent women, or 32 births for every 10,000. Adult fertility and youth homicide rate also had an independent positive association with adolescent fertility. Model 3 included state-level income inequality which was not significantly associated with adolescent fertility. Models 4 to 6 followed the same sequence for the year 2010, and found similar results for the association between municipal income inequality and adolescent fertility. The difference between the IGLS deviance results indicate that the models with all the variables (Models 3 and 6) provided a better overall fit to the data. Model 7 includes the results for both years simultaneously (2000 and 2010), which means that despite having a larger IGLS deviance, this result is not directly comparable with the other models. Model 7 presents the coefficient of the interaction terms for 2010 for each of the covariates. The result from the interaction term between the Gini Coefficient and the dummy for the year 2010 indicates that the association between income inequality and adolescent fertility did not change significantly between the year 2000 and 2010 (p > 0.05).

**Table 2:** **Multilevel regression models for the association between adolescent fertility and explanatory variables**

	**2000**					
	**Model 1**		**Model 2**		**Model 3**	
	**B**	**95% CI**	**B**	**95% CI**	**B**	**95% CI**
Intercept	4.37*	2.81; 5.92	0.83	-0.31; 1.79	0.74	-1.75; 3.23
Gini Coefficient	6.61*	3.62; 9.61	3.32*	1.76; 4.70	3.18*	1.49; 4.88
Per capita income (R$)			0.00		0	
Adult fertility^a^			0.94	0.88; 1.00	0.94*	0.88; 1.00
Mothers with 12 years or more of formal education (%)			-0.01	-0.03; 0.01	-0.01	-0.03; 0.01
Youth homocide rate^b^			0.07*	0.02; 0.12	0.07*	0.01; 0.12
State-level inequality					0.07	-4.21; 4.55
IGLS Deviance	30410.84		27177.34		27177.32	

^a^Ratio between the number of live births from women aged 20 to 49 and the number of women aged 20 to 49.

^b^Homicide rate (per 10,000) of 10 to 19 years old residents.

*p < 0.05.

Brazilian municipalities, 2000.

**Table 3:** **Cross-sectional multilevel regression models for the association between adolescent fertility and explanatory variables**

	**2010**						**2010 + 2010**	
	**Model 4**		**Model 5**		**Model 6**		**Model 7**	
	**B**	**95% CI**	**B**	**95% CI**	**B**	**95% CI**	**B**	**95% CI**
Intercept	3.19*	1.46; 4.81	0.14	-0.40; 0.68	1.23	-0.01; 2.48	0.45	-2.30; 3.21
Gini Coefficient	5.56*	2.19; 8.93	3.46*	2.25; 4.68	4.30*	3.14; 5.45	1.11	-0.94; 3.16
Per capita income (R$)			0.00	-	0.00	-	0.00	-
Adult fertility^a^			1.06*	0.99; 1.13	1.06*	0.99; 1.13	0.13	0,04; 0.21
Mothers with 12 years or more of formal education (%)			-0.02	-0.03; 0.08	-0.02	-0.03; 0.01	-0.01	-0.03; 0.01
Youth homocide rate^b^			0.06*		0.06*	0.04; 0.08	-0.01	-0.06; 0.05
State-level inequality					-2.51*	-4.74; -0.28	-2.59	-7.47; 28
IGLS Deviance	28263.03		23705.90		23695.37		51365.22	

^a^Ratio between the number of live births from women aged 20 to 49 and the number of women aged 20 to 49.

^b^Homicide rate (per 10,000) of 10 to 19 years old residents.

*p < 0.05.

Brazilian municipalities, 2010 and 2000 + 2010.

Table 4 presents the results for the longitudinal first-differences multilevel models. It shows that a change in income inequality was significantly associated with higher adolescent fertility, and remained so after the inclusion of the control variables. Model 3 indicates that a longitudinal increase of 0.1 in the Gini Coefficient was associated with a longitudinal increase of 0.41 live births for every 100 adolescent woman. As in the cross-sectional models, adult fertility was also positively associated with adolescent fertility, indicating the presence of an overall effect of increased fertility in adolescent fertility. Contrary to the previous models, however, youth homicide rate was not associated with increased adolescent fertility. State-level inequality was also not associated with adolescent fertility.

**Table 4:** **Longitudinal first-differences multilevel models for the association between adolescent fertility and explanatory variables**

	**Model 1**		**Model 2**		**Model 3**	
	**B**	**95% CI**	**B**	**95% CI**	**B**	**95% CI**
Intercept	-1.84	-1.97; 1.70	0.2*	0.02; 0.39	0.20	-0.02; 0.43
Gini Coefficient	4.42*	2.79; 6.05	4.08*	2.82; 5.33	4.08*	2.77; 5.38
Per capita income (R$)			0.00	-	0.00	-
Adult fertility^a^			0.96	0.92; 1.01	0.96*	0.92; 1.01
Mothers with 12 years or more of formal education (%)			-0.02*	-0.03; -0.01	-0.02*	-0.03; -0.01
Youth homocide rate^b^			0.00		0.00	-
State-level inequality					-0.02	-4.68; 4.63
IGLS Deviance	28515.68		25351.48		25351.48	

^a^Ratio between the number of live births from women aged 20 to 49 and the number of women aged 20 to 49.

^b^Homicide rate (per 10,000) of 10 to 19 years old residents.

*p < 0.05.

Brazilian municipalities, 2000 + 2010.

We then introduced a sensitivity analysis to check for the robustness of the results, by including only municipalities with satisfactory birth record coverage (Table 5). The longitudinal association between income inequality and adolescent fertility remained positive and significant for all of the three models tested. For Model 3, a longitudinal increase of 0.1 in the Gini Coefficient was associated with a longitudinal increase of 0.25 live births for every 100 adolescent women. Similarly, adult fertility remained significantly associated, while youth homicide rate was not. We also analyzed the null models of each of the dependent variables to assess how much of the variance could be explained by each of the two levels. In the year 2000, 47.8% of the variance was explained by the second level and 52.2% by the first. For 2010, the percentage was 53.3% and 45.7%, respectively. For the first differences longitudinal model, 17.8% of the variance was explained by the second level and 82.2% by the first level.

**Table 5:** **Longitudinal first-differences multilevel models for the association between adolescent fertility and explanatory variables for municipalities with satisfactory birth coverage (n = 2,436)**

	**Model 1**		**Model 2**		**Model 3**	
	**B**	**95% CI**	**B**	**95% CI**	**B**	**95% CI**
Intercept	-2.27*	-2.51; -2.03	-1.30*	-1.66; -0.93	-1.31*	-1.67; 0.95
Gini Coefficient	4.40*	1.28; 7.52	2.40*	0.23; 4.58	2.31*	0.15; 4.94
Per capita income (R$)			0.00	-	0.00	-
Adult fertility^a^			0.58*	0.50; 0.68	0.58*	0.50; 0.67
Mothers with 12 years or more of formal education (%)			-0.02*	-0.03; -0.01	-0.02*	-0.03; 0.01
Youth homocide rate^b^			-0.02	-0.05; 0.01	-0.02	-0.5; 0.01
State-level inequality					-0.70	-6.39; 4.99
IGLS Deviance	10638.88		10120.64		10120.47	

^a^Ratio between the number of live births from women aged 20 to 49 and the number of women aged 20 to 49.

^b^Homicide rate (per 10,000) of 10 to 19 years old residents.

*p < 0.05.

Brazilian municipalities, 2000 + 2010.

## Discussion

Our results indicate a consistent and positive association between income inequality and adolescent fertility. We first tested this association cross-sectionally and then fitted a first-differences longitudinal model to control for time-constant effects, with similar results. For the final first-differences model, a longitudinal increase of 0.10 in the Gini Coefficient was associated with a longitudinal increase of 0.41 live births for every 100 adolescent mothers. Adult fertility (from women aged 20 to 49 years old) was also positively associated with adolescent fertility for every model. Results for youth homicide rate were mixed. Finally, per capita income does not seem to be associated with adolescent fertility.

A number of authors have suggested that when there is a perception of future economic advancement, either due to a more equal or mobile society, adolescents have an incentive to delay motherhood and invest in human capital, decreasing adolescent pregnancies [4,5]. Adolescent fertility could also be influenced by adolescent men’s perspectives, an area of research that has not been sufficiently addressed by the literature, especially regarding socioeconomic differences in expectations and decision-making [21]. Previous studies that analyzed income inequality and adolescent pregnancies focused solely on cross-sectional analyses of developed countries. Our study longitudinally analyzed a large number of municipalities in a developing country with a very high level of income inequality.

Adolescent pregnancies have been associated with a number of adverse future socioeconomic outcomes for the mother, such as a lower probability of receiving high school diploma, lower income as a young adult and a higher probability of receiving cash assistance [22]. Adolescent pregnancies have been suggested to create an infinite loop of poverty, where adverse social conditions increase adolescent pregnancies which itself leads to worse social conditions.

Previous studies have mostly analyzed individual risk factors for adolescent fertility. Recent studies in Brazil have suggested that individual risk factors may have lost at least some of its influences. In a recent Brazilian study of teenage girls from the municipality of São Paulo, most of them answered positively when asked if one should always wear a condom when having sexual relationships, a proportion that did not change whether the teenager was from a public (96.6%) or private school (96.7%) [23]. Another study of adolescent mothers in a poor area of Brazil also indicated that adolescents had adequate knowledge about contraceptive methods and about the consequences of unprotected sex [24].

Our results suggest the existence of an effect of broader, contextual factors on adolescent fertility. Besides income inequality, adult fertility was also independently and positively associated with adolescent fertility. This suggests that adolescent fertility and adult fertility are likely to move in the same direction, indicating the presence of a local peer effect on motherhood, as found by Vundule et al. [18].

Youth homicide rate was cross-sectionally associated with adolescent fertility, an association also previously reported by Pickett et al. [12] on a sample of developed countries and US states. However, when we fitted the longitudinal models, this association was no longer significant. This could be explained by the confounding effect of time-constant variables such as area-level institutional presence and law enforcement, which influence both homicide and birth rate (as abortions, a common plan B for adolescents, are illegal in Brazil). The use of longitudinal models controls for time-constant variables, which suggests that there is no direct association between youth homicide rate and adolescent fertility.

Our results also indicate that the association of income inequality on adolescent fertility happens primarily on the municipality-level. State-level income inequality was not significant in any of the models. As most of the previous studies that analyzed the effects of inequality in the US focused on state-level differences, our results suggest that by not including municipalities the association between inequality and health outcomes could have been underestimated.

The present study adds to a growing literature on how social determinants affect the health of populations. It is well established that some social characteristics such as poverty and education are associated with a large number of health outcomes, but the effect of income inequality has only recently entered the public debate in developed countries, especially after the analyses of Wilkinson & Pickett [25] that showed that unequal countries frequently lag behind on a variety of health problems. The study of the social determinants of health has the potential of identifying important conditions that directly affect the health of populations and of being a propeller for social and economic change.

Our analyses focused solely on the effect of income inequality on live births. Another possible effect of income inequality would be on the abortion rate of the municipalities. In the case of Brazil, that analysis is still not possible, as the great majority of abortions are illegally induced and thus not reported by municipalities or states [26]. The possibility of a direct effect of income inequality on abortions could influence our results, as in the cases of more unequal areas having a lower number of abortions. As a historically religious country, Brazil still has a complex relationship with abortions, and the magnitude of the problem is still largely unknown. The emergence of field studies that could analyze this relationship is warranted.

The study has a few other limitations. First, Brazil is a large and heterogeneous country, where the quality of the data differs significantly between the regions. We tried to account for that by adding an analysis that included only municipalities with satisfactory live birth coverage, but other biases may remain. Second, some of the determinants of adolescent fertility may be clustered within smaller areas than municipalities, such as neighborhoods, for which data is not available. Third, the study relied on secondary data that can be subject to measurement errors, especially in very small and poor municipality where per capita income is unclear due to informal work and where homicides are underreported. Fourth, the direct generalization of our finds to other countries should be handled with caution given the particular socioeconomic pathway that Brazil went through during the period (increased income and education with decreased inequality).

## Conclusion

In developing countries, complications from pregnancy and childbirth are the leading cause of death for women aged 15 to 19 [27]. It is estimated that each year 16 million adolescents become mothers, representing 11% of all births worldwide, with 95% of these occurring in developing countries [28]. Our study found that from the year 2000 to 2010, income inequality was an important determinant of adolescent fertility in Brazil. This result is consistent with previous theories regarding a “culture of despair”, where an early motherhood is a rational response to a society that doesn’t offer many other options of socioeconomic advancement. Understanding and addressing the consequences of income inequality is expected to be a leading concern for most countries in the near future. Our results suggest that adolescent pregnancies should be included in the list of health outcomes that are influenced by income inequality.
